# Supplementary material for: Drug repurposing for aging research using model organisms
Source: Aging Cell. 2017 Jun 16;16(5):1006–15. doi: 10.1111/acel.12626 (PMC5595691; doi:10.1111/acel.12626)
Supplement: Supplementary file 7 — Data S1 Zip‐Archive of all report cards. [file ACEL-16-1006-s007.zip › RC_BHF.pdf]

BHF

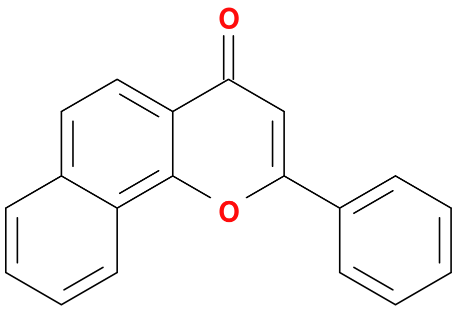

Database identifiers

ChEMBLCompound	CHEMBL283196
DrugBank	DB07453
CHEBI	76995
ZINC	ZINC00038933
eMolecules	499134

Ranking

	Rank	Score
Drosophila	430/697	0.334
C. elegans	NA	NA

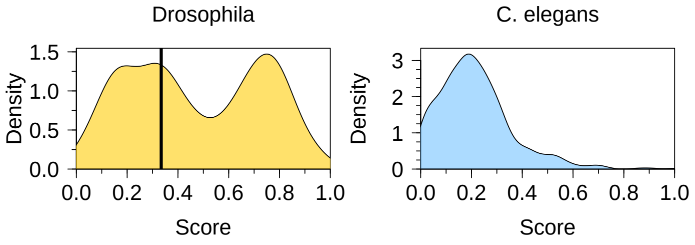

	Ageing implication	Domain conservation	Binding site conservation	Binding affinity	Bioavailability	Lipinski	Promiscuity	Purchasability	Drug approval	**Total**
Drosophila	0.792	0.613	0.413	0.884	(0.9)	0.0	-0.0	0.1	0.075	0.334
C. elegans	NA	NA	NA	NA	NA	NA	NA	NA	NA	NA

Names

- **alpha-naphthoflavone**
- 2-PHENYL-4H-BENZO[H]CHROMEN-4-ONE
- 2-phenyl-4H-naphtho(1,2-b)pyran-4-one
- 7,8-Benzoflavone
- 7,8-bf
- alpha-naphthylflavone
- benzohflavone

Roles

EC 1.14.14.14 (aromatase) inhibitor, aryl hydrocarbon receptor antagonist, aryl hydrocarbon receptor agonist

Status

Approved drug (according to ChEMBL)	No
Classification (according to DrugBank)	experimental
Number of Rule of 5 violations	0
Binding affinity to original target in log units (RF-Score prediction)	7.03
Burns *C. elegans* bioavailability prediction	8.37

Compound Target Characteristics

Cytochrome P450 1A1

Best gene implication in ageing for this target family came from gene P00185 annotated in UniProt release 2014_02. Annotation GO 7568 (aging) was Inferred from Expression Pattern

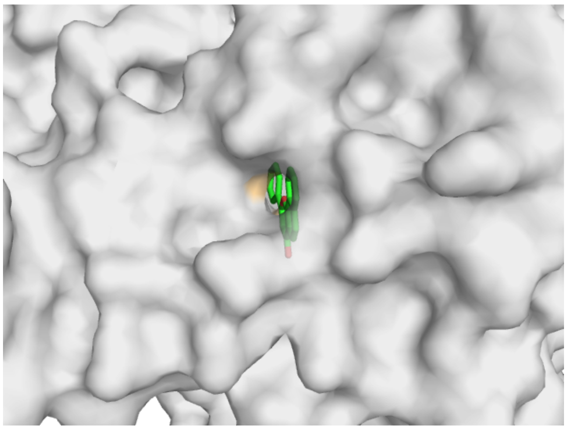

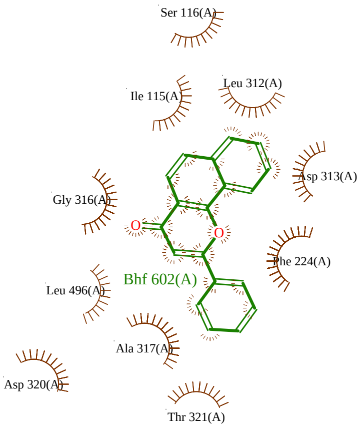

protein	amino acids contacts (binding site)
PDB:4i8v:chainA:P04798	I S N F L D G A D T L
tr:E7EMT5:E7EMT5_HUMAN	I S N F L D G A D T L
tr:A0N0X8:A0N0X8_HUMAN	I S N F L D G A D T L
sp:P04798:CP1A1_HUMAN	I S N F L D G A D T L
sp:P00185:CP1A1_RAT	I A N F F D G A D T L
sp:P00184:CP1A1_MOUSE	I T N F L D G A D T L
tr:Q05A20:Q05A20_MOUSE	I T N F L D G A D T L
sp:Q9VRM7:CP307_DROME	L F D I E D G - S A L
tr:H8F4V5:H8F4V5_DROME	L F D I E D G - S A L
tr:A8Y592:A8Y592_DROME	L F D I E D G - S A I

	whole protein		domain-based		contact-based	
protein	ident	simil	ident	simil	ident	simil
PDB:4i8v:chainA:P04798	1.0	1.0	1.0	1.0	1.0	1.0
tr:E7EMT5:E7EMT5_HUMAN	0.94	0.94	0.94	0.94	1.0	1.0
tr:A0N0X8:A0N0X8_HUMAN	1.0	1.0	1.0	1.0	1.0	1.0
sp:P04798:CP1A1_HUMAN	1.0	1.0	1.0	1.0	1.0	1.0
sp:P00185:CP1A1_RAT	0.78	0.92	0.81	0.95	0.82	0.89
sp:P00184:CP1A1_MOUSE	0.78	0.92	0.81	0.95	0.91	0.95
tr:Q05A20:Q05A20_MOUSE	0.78	0.92	0.81	0.95	0.91	0.95
sp:Q9VRM7:CP307_DROME	0.25	0.65	0.26	0.65	0.27	0.41
tr:H8F4V5:H8F4V5_DROME	0.25	0.65	0.26	0.65	0.27	0.41
tr:A8Y592:A8Y592_DROME	0.23	0.63	0.23	0.64	0.18	0.41

spo (UniProt:Q9VRM7) annotation

Function: Required for correct development of the embryonic midline glial cells which are necessary for the formation of distinct segmental commissures. (PubMed:10328928).
Cofactor: heme
Subcellular location: Endoplasmic reticulum membrane ; Peripheral membrane protein Micro-

some membrane ; Peripheral membrane protein
Miscellaneous: Member of the Halloween gene group.

(Information from UniProt)

spok (FBgn0086917) associated phenotypes

lethal - all die before end of first instar larval stage, some die during first instar larval stage

(Information from FlyBase)
